# Will tomorrow's mineral materials be grown?

**DOI:** 10.1111/1751-7915.14298

**Published:** 2023-07-31

**Authors:** Julie Cosmidis

**Affiliations:** ^1^ Department of Earth Sciences University of Oxford Oxford UK

## Abstract

Biomineralization, the capacity to form minerals, has evolved in a great diversity of bacterial lineages as an adaptation to different environmental conditions and biological functions. Microbial biominerals often display original properties (morphology, composition, structure, association with organics) that significantly differ from those of abiotically formed counterparts, altogether defining the ‘mineral phenotype’. In principle, it should be possible to take advantage of microbial biomineralization processes to design and biomanufacture advanced mineral materials for a range of technological applications. In practice, this has rarely been done so far and only for a very limited number of biomineral types. This is mainly due to our poor understanding of the underlying molecular mechanisms controlling microbial biomineralization pathways, preventing us from developing bioengineering strategies aiming at improving biomineral properties for different applications. Another important challenge is the difficulty to upscale microbial biomineralization from the lab to industrial production. Addressing these challenges will require combining expertise from environmental microbiologists and geomicrobiologists, who have historically been working at the forefront of research on microbe–mineral interactions, alongside bioengineers and material scientists. Such interdisciplinary efforts may in the future allow the emergence of a mineral biomanufacturing industry, a critical tool towards the development more sustainable and circular bioeconomies.

## Graphical abstract

Microbial biominerals possess specific and original mineralogical properties that make then promising materials for a range of technological applications. The future development of these applications will require us to decipher the genetic and biomolecular mechanism at the origin of the microbial “mineral phenotype”.

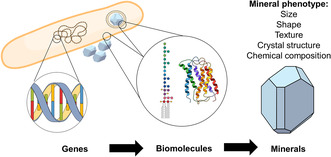

## INTRODUCTION

There is an old adage in the mining industry: ‘if it can't be grown, it has to be mined’. Look around you, and you will probably see more objects made from materials extracted from Earth's crust than materials grown by living beings at its surface. The world's appetite for minerals and metals is stronger than ever and is expected to at least double by 2060 (OECD, 2019). Amazingly, since 2020, the total mass of ‘things’ made by humans from mined materials actually surpasses the global living biomass (Elhacham et al., 2020). Expanding cities require enormous amounts of raw materials such as lime, sand or iron, for the production of cement and steel, two of the ‘four pillars of modern civilization’ (Smil, 2022). But more than raw minerals and metals, a specificity of the modern age is the need for advanced, engineered materials such as those making up our batteries and photovoltaic cells, but also industrial catalysts, sensors or high‐tech biomedical devices. Advanced technological materials have well‐defined physical, chemical, magnetic, optical or electronical properties, and their design is the focus of much of today's material engineering efforts. But could biology help too?

In this opinion piece, I discuss the idea that microbes could in the future be used to engineer and manufacture advanced mineral materials. Bacteria are already supporting industry in a multitude of processes, from the traditional use of fermentation products in food making, to modern, genetically engineered ‘microbial cell factories’ producing pharmaceuticals, biofuels, biocatalysts and other high‐value chemicals (Singh, 2021). In recent years, there has been growing interest in the use of bacteria and fungi for the synthesis of polymeric organic materials such as microbial cellulose or mycelium, which may one day replace polymers derived from the petrochemical industry in everyday objects (e.g. Hernández‐Arriaga et al., 2022; Martirani‐VonAbercron & Pacheco‐Sánchez, 2023; Melton, 2022; Meyer et al., 2020). There is no doubt that in the next decades, synthetic biology and microbial biomanufacturing will become critical tools to help us meet our needs for chemicals and materials and build more sustainable and circular bioeconomies (Antranikian & Streit, 2022). But there is no reason to limit these efforts to organic products. In addition to organic molecules and polymers, many bacteria possess the capacity to produce minerals, a process called biomineralization. So far, microbial biomineralization has been predominantly studied by environmental microbiologists and geomicrobiologists interested in understanding the impact of microbes in mineral formation processes in nature. But microbial biomineralization also holds a widely untapped potential for the design of new mineral materials. Engineered bacteria may in the future biomanufacture advanced minerals for industry, in a way that is likely to be more energy efficient and environmentally friendly than traditional processes based on chemical or metallurgical synthesis. Although we may still be decades away from fully realizing this potential, here I try to highlight the main roadblocks along the way and propose some clues as to how they may be lifted.

## BACTERIA ARE NATURAL MATERIAL ENGINEERS

Biominerals are a fascinating testimony of life's capacity to shape the inorganic world. From the extracellular carbonates precipitated by stromatolite‐building cyanobacteria (Dupraz et al., 2009), to the intracellular iron minerals used by magnetotactic bacteria to navigate along Earth's magnetic field lines (Faivre & Schüler, 2008), biomineralization has evolved in a wide diversity of microbial lineages as an adaptation to a range of environmental conditions and biological functions (Cosmidis & Benzerara, 2022). Through the formation of minerals, microbes participate in transformations and fluxes of most elements present at the surface of the planet (Falkowski et al., 2008), in a process that is increasingly regarded as an important driving force in life's long coevolutionary history with Earth's surface environments (Williams & Rickaby, 2012).

Microbial biomineralization mirrors eukaryotes' capacity to produce mineral skeletons, teeth and shells. However, prokaryotic biomineralization encompasses a much wider range of chemistries and mineral types, mostly due to the impressive versatility of microbial metabolisms (biominerals are in many cases composed of chemical end‐products of metabolic pathways; Hoffmann et al., 2021). In addition to carbonates (Görgen et al., 2021), phosphates (Fishman et al., 2018), silica (Li et al., 2022) and iron oxides (Faivre & Schüler, 2008), biomineral types that are shared with Eukaryotes, microbes capable of biomineralizing industrially relevant metals as diverse as uranium (Wufuer et al., 2017), tellurium (Baesman et al., 2007), selenium (Nancharaiah & Lens, 2015), palladium (Egan‐Morriss et al., 2022), gold (Fairbrother et al., 2013; Gwynne, 2013), silver (Ramalingam et al., 2014), copper (Pantidos et al., 2018; Park et al., 2022) or rare‐earth elements (Maleke et al., 2019) have now been described, and many more remain to be discovered.

Similar to eukaryotic biominerals, minerals formed by bacteria often possess unique properties such as non‐crystallographic morphologies, thermodynamically unstable structures, out‐of‐equilibrium chemical compositions and intimate association with organic matrices. From an engineering perspective, these original properties of biominerals would often be difficult to reproduce through chemical synthesis in a laboratory or an industry setting. Furthermore, biomineralizing bacteria can often form minerals under thermodynamically unfavourable conditions and kinetically outcompete chemical mineral precipitation in Earth's surface environments (e.g., Ferris et al., 2016; Luther et al., 2011). On the other hand, extremophiles capable of biomineralization under harsh temperature or pH conditions, and/or in the presence of high concentrations of toxic metals, make microbial biomineralization a potentially viable process for mineral manufacturing under a wide range of industrially relevant conditions (Ehrlich et al., 2021).

Harnessing the biomineralization capabilities of bacteria thus appears an attractive pathway to manufacture high‐value, difficult‐to‐obtain materials with unique properties for diverse technological applications. Compared with conventional processes, mineral bioproduction may in many cases save energy and resources and reduce carbon emissions (especially when combined with waste biorecycling, critical metal biorecovery, or CO_2_ fixation; e.g., He & Kappler, 2017; Kajla et al., 2022; Macaskie et al., 2010; Patel et al., 2021) and limit the need for toxic chemicals and harsh physicochemical conditions. As described in the section below, material applications of microbial biomineralization have already been developed with some success for a limited number of biomineral types.

## CURRENT AND POTENTIAL APPLICATIONS OF BIOMINERALIZED MATERIALS

Among the applications of microbial biomineralization that are currently being developed, the most mature is without doubt the use of magnetic iron nanoparticles, which are formed intracellularly by magnetotactic bacteria. These biogenic magnetite (Fe_3_O_4_) or greigite (Fe_3_S_4_) crystals display critical properties that distinguish them from abiotically formed counterparts, such as high chemical purity, few crystal defects, and, most importantly, restricted size and shape ranges so as to form stable single‐magnetic domains (Amor et al., 2020). Biogenic magnetic nanoparticles are also encapsulated within a lipidic membrane, forming an organelle called the magnetosome, which can now be functionalized for the surface display of fluorescent tags, antibodies or biocatalysts (Mickoleit et al., 2020; Mittmann et al., 2022; Xu et al., 2019). Magnetosomes can easily be isolated from lysed cells, enabling their use in a number of applications, mostly in the biomedical field (e.g. in magnetically targeted drug delivery, pathogen detection, magnetic hypothermia for the treatment of tumours, or as contrast agents in magnetic imaging techniques), but also for toxic metal bioremediation or the detection of pathogens in food – applications that are described in greater details in a number of recent reviews (Basit et al., 2020; Kotakadi et al., 2022; Qin et al., 2020; Vargas et al., 2018). Other types of microbially formed metal nanoparticles (e.g. Au, Ag, Cu, Pd, Mn) are now being considered for a wide range of industrial uses including water remediation, electrochemical energy storage, catalysis, and biomedical applications such as antimicrobial activity, bioimaging and biolabeling (e.g. Egan‐Morriss et al., 2022; Galezowski et al., 2023; Qin et al., 2020). Biomineralization of metallic nanoparticles presents several advantages over chemical or physical methods, such as improved particle stability and biocompatibility, as well as reduced environmental impact (Atalah et al., 2022; Carmona et al., 2023).

Manufacturing of construction materials is another field where applications of microbial biomineralization are being intensively researched. In particular, calcium carbonate materials precipitated by ureolytic or photosynthetic bacteria are being considered as alternatives to Portland cement concrete (Beatty et al., 2022). This biological approach has the potential to significantly reduce the enormous environmental impact of concrete production, which is currently the second largest industrial CO_2_ emitter globally (International Energy Agency, 2018). In addition to improved sustainability, biomineralized construction materials have the potential to display longer life times as compared to conventional concrete due to self‐healing properties, which may be achieved by maintaining viable biomineralizing bacteria within the material itself (Jonkers, 2007). However, if the production of such ‘living building materials’ (Heveran et al., 2020; Qiu et al., 2021) is relatively straightforward to achieve at the laboratory scale, the industrial deployment of microbial biomineralization for construction is facing important challenges such as low scalability and high costs (Beatty et al., 2022). With about 30 billion tonnes of concrete consumed globally each year (slightly less than 4 tonnes per living person on Earth) (Monteiro et al., 2017), significant progress would have to be made on these two fronts before microbes can make even a small contribution to the transformation of the construction industry.

More generally, the difficulty to cost‐effectively upscale microbial biomineralization is certainly the biggest hurdle along the way towards mass bioproduction of mineral materials. However, pilot‐plant scale production has now been achieved for several types of microbial biominerals (Moon et al., 2010, 2013, 2016), bringing some hope that microbial biomineralization processes may not be trapped forever in what is known by venture capitalists as the ‘valley of death’ between discovery and commercialization. A possibly more recalcitrant issue, detailed below, probably relies in our incapacity to engineer most biomineralization processes.

## TOWARDS MINERAL BIOENGINEERING?

In order to perform in technological application, properties of microbial biominerals such as particle size, crystal structure or chemical composition may need to be improved and fined‐tuned. In principle, the fantastic advances in genome‐editing techniques (including CRISPR technologies; Wang & Doudna, 2023) should allow us to design genetically engineered microbial strains with optimized biomineralization capabilities for diverse applications. But in reality, biomineral engineering has only rarely been achieved so far. This is primarily due to the fact that for the vast majority of microbial systems, we still have a very limited understanding of the intricate genetic and biomolecular mechanisms controlling biomineralization. Magnetotactic bacteria are a notable exception, for which our in‐depth knowledge of the genetic determinants of biomineralization has enabled the development of bioengineering approaches aiming at altering, for instance, the morphology of magnetite crystals (Yamagishi et al., 2016) or the configuration of the chain formed by magnetosomes inside the cell (Amor et al., 2022). It is now even possible to entirely engineer magnetosome production in originally non‐magnetic bacteria through the transfer and expression of synthetic magnetosome‐encoding gene clusters (Mickoleit et al., 2021). Other attempts to encode biomineralization capacities into non‐biomineralizing bacteria have typically been limited to the heterologous expression of recombinant enzymes such as the urease (which induces carbonate precipitation following ureolysis‐driven pH increase; e.g. Bachmeier et al., 2002) or the phosphatase (which induces phosphate precipitation following the release of organically bound phosphorus; e.g. Cosmidis et al., 2015). While these approaches are relatively efficient to obtain large amounts of minerals from easy‐to‐grow strains such as *E. coli*, they offer poor control on the mineralogical properties of the resulting products. Although it is possible to slightly alter the properties of the precipitated minerals by changing the cellular location of the expressed enzyme or its activity level (e.g. Cosmidis et al., 2015; Heveran et al., 2019), these approaches cannot be used to deliberately design novel advanced mineral materials with well‐defined properties, such as high chemical purity, organic encapsulation, specific crystal structures or restricted size distributions and morphologies. Doing so would require gaining a much better understanding of the biological mechanisms controlling all these mineral properties. In other words, the development of mineral bioengineering strategies requires that we gain a much deeper knowledge of the genetic determinants of the ‘mineral phenotype’, a concept that is further developed below.

## WHAT IS THE ‘MINERAL PHENOTYPE’ AND WHAT CONTROLS IT?

**FIGURE 1:**
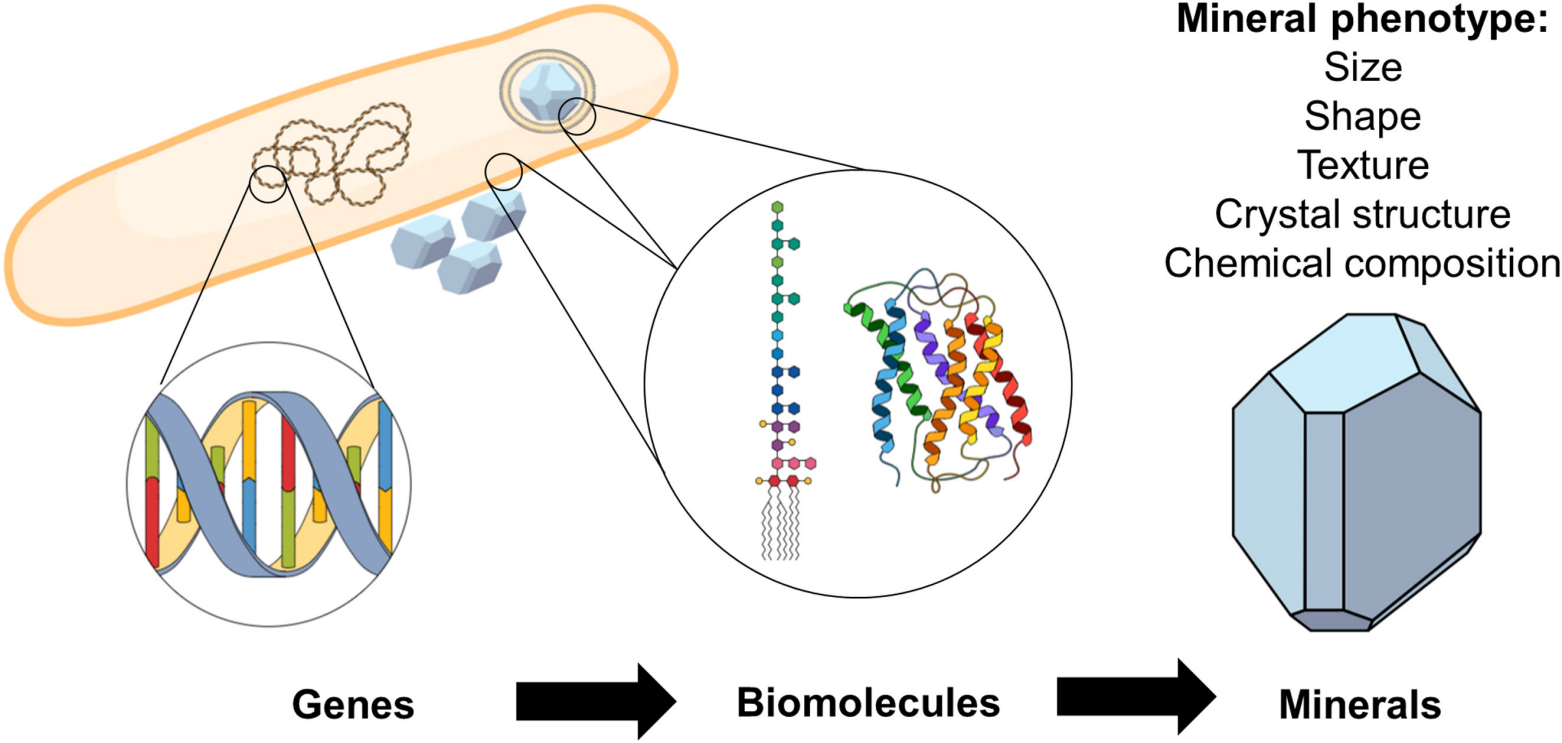
The specific properties of adaptive microbial biominerals are controlled by biomolecules directing mineral formation (e.g. forming the vesicles within which the biominerals form, or the surfaces on which they nucleate). These biomolecules (e.g. proteins, lipids, polysaccharides) are encoded in the genome. For most microbial biomineralization systems, the genetic and biomolecular controls of the mineral phenotype are still poorly understood, limiting our ability to rationally engineer microbial biominerals for specific applications.

As a general rule, microbial biominerals can be classified into two categories. The first one consists in minerals that are formed as by‐products of metabolic activity and that are not assumed to have any biological function other than ‘waste’. Typically, these minerals are formed extracellularly, and their mineralogical properties are determined by the geochemical environment rather than encoded in the bacterial genome. The second category consists in biominerals that contribute to the organism's fitness by playing important biological roles such as protection, nutrient or energy storage (in the form of electron donors or acceptors for metabolism), navigation, motility, detoxification, extracellular electron transfer or participation in biofilm architecture (Cosmidis & Benzerara, 2022; Phoenix & Konhauser, 2008). These ‘adaptive’ biominerals typically have mineralogical properties that strikingly differ from those of inorganically precipitated counterparts, properties that are genetically encoded and tightly controlled by the microbial cell. The ensemble of these properties composes the ‘mineral phenotype’ (Figure 1).

Gaining a further understanding of the genetic and biomolecular controls of the mineral phenotype is a prerequisite of biomineral engineering efforts. However, very little is known about the underlying molecular mechanisms of most microbial biomineralization pathways. In the best studied example of adaptive biomineralization, magnetotactic bacteria, dozens of different proteins are required to direct the nucleation and growth of the iron biominerals inside the magnetosomes and to align them into a chain within the cell (Amor et al., 2020; McCausland & Komeili, 2020; Uebe & Schüler, 2016). This complex machinery is encoded by genes clustered in a region of the bacterial chromosome called the magnetosome island, which typically spreads over ~2% of the bacterial genome. For other bacterial systems, such a fine level of understanding of the genetic basis of the mineral phenotype is still out of reach. It is safe to write that most of the genes and biomolecules controlling microbial biomineral properties in nature remain to be discovered.

The increasingly more widespread use of next‐generation DNA sequencing techniques in recent years has clearly enabled important progress in our understanding of the genetic basis of microbial biomineralization. We have learned about specific genes associated with the formation of different biominerals and discovered how they are distributed in the environment and how they may interact in complex networks in ecosystems. However, there has been a strong bias towards the study of metabolic genes involved, for instance, in energy‐generating oxidation–reduction processes such as sulphur‐oxidation (Dahl, 2020), iron‐oxidation (Kappler et al., 2021) or coding for chemistry‐altering enzymes such as ureases (e.g. Ferrer et al., 2021) or phosphatases (e.g. Skouri‐Panet et al., 2018). These metabolic reactions are often the primary drivers of microbially induced mineral precipitation, but their role is primarily catalytic. Indeed, they mainly control the kinetics of the mineral formation reaction, for instance, by increasing the rate at which soluble Fe(II) is oxidized to insoluble Fe(III) by oxygen (Emerson et al., 2010). Although in some cases these catalytic effects may influence mineral properties (for instance, high supersaturations resulting from enzymatically driven redox reactions are thought to induce the precipitation of exceptionally small mineral particles; Banfield et al., 2001), the genetic‐ and molecular‐level mechanisms controlling phenotypic characteristics of biominerals such as their morphology or crystal structure remain vastly unexplored. This limited knowledge makes it difficult to predict the effect of genetic mutations on biomineralization processes and resulting mineral phenotypes and limits the ability to rationally design mutations that improve biomineral properties for different applications.

## THE SCREENING BOTTLENECK

As shown by the example of magnetotactic bacteria, adaptive biomineralization is typically a complex trait, involving a great number of genes acting together to produce biominerals with specific properties. Gaining control on the properties of engineered biominerals requires us to elucidate these intricate biomolecular systems. This is not an essentially intractable problem. Modern microbiology approaches now allow us to improve our understanding of the genetic basis of phenotypic variations, that is, to determine how changes to encoding DNA sequences can alter gene functions and phenotypes (Kobras et al., 2021). A typical approach, when working with a cultivable microbial strain, consists in generating a library containing hundreds or thousands of mutants in the laboratory and then screening this library in order to identify the mutants displaying alterations of the phenotype of interest. Comparing the genomes of the mutants with that of the original strain, mutations can be located, and genes essential to this phenotype can be identified. Mutagenesis followed by screening has proven a very powerful to elucidate the genetics of magnetosome formation as early as the 1990s and early 2000s (e.g. Matsunaga et al., 1992; Wahyudi et al., 2001), but it has yet to be deployed on other microbial biomineralization system. So, why only magnetotactic bacteria? It goes without saying, but magnetotactic bacteria are magnetic. For this reason, non‐biomineralizing mutants can easily be separated from biomineralizing ones by application of an external magnetic field (McCausland & Komeili, 2020). Magnetotactic bacteria are thus very conveniently encoding their own screening tool. For other types of biomineralizing bacteria, we still lack the tools that would allow us to scan hundreds of mutant strains at a time and discriminate them based on their biomineralization capacity and mineral phenotype. Analytical methods for the identification and characterization of biominerals classically include approaches such as electron microscopy, X‐ray diffraction or X‐ray spectromicroscopy (e.g. Cosmidis & Benzerara, 2014; Miot et al., 2014). These methods cannot be used to process large numbers of samples at a time and, importantly, they are destructive and cannot be applied directly to liquid or solid cultures. They furthermore require labour‐ and time‐intensive sample preparation steps. Flow sorting methods through differential light‐scattering or fluorescence (e.g. Bawazer et al., 2012) could in principle be employed to separate biomineralizing from non‐biomineralizing bacteria. However, they cannot provide fundamental mineralogical information such as chemical composition, particle morphology, surface properties or crystal structure‐information that would be needed to discriminate mutants based on their biomineral properties.

The lack of an appropriate screening tool is thus arguably the main bottleneck slowing down the application of mutagenesis approaches to the elucidation of the mineral phenotype. Raman spectromicroscopy may in the future form an ideal foundation to build such a tool. Raman is a vibrational spectroscopy technique, which can provide information on a sample's chemical composition and crystal structure. Coupled with optical or confocal microscopy, Raman can be used to image and analyse objects smaller than one micrometre. Raman does not require any sample preparation and can be performed at room temperature and atmospheric pressure, so that wet or liquid samples can be characterized. Furthermore, the laser excitation used to generate the Raman signal is non‐destructive to cells, enabling in vivo analyses (Lee et al., 2021). Raman has already been used to provide chemical and structural information on a range of (bio)minerals (Nasdala & Schmidt, 2020; Steele et al., 2020), including on live microbial cultures (Nims et al., 2019), and high‐throughput screening Raman spectroscopy systems already employed by biochemists (Kawagoe et al., 2021; Kojima et al., 2010) or cell biologists (Schie et al., 2018) could probably be adapted to screen microbial mutant strains separated in multi‐well plates. There is thus hope that in the future, the development and application of high‐throughput mineral characterization techniques combined with next‐generation sequencing techniques will enable significant progress in our understanding of the intricate molecular controls of microbial biomineralization processes.

## IDENTIFYING NEW GENES ASSOCIATED WITH BIOMINERALIZATION IN ENVIRONMENTAL STRAINS

Mutagenesis is not the only way we can learn about the genetic controls of microbial biomineralization. In recent years, different research groups have successfully employed comparative genomics to identify candidate genes associated with, for instance, extracellular iron mineral formation by microaerophilic iron‐oxidizing bacteria (Kato et al., 2015; Koeksoy et al., 2021), or intracellular carbonates biomineralization in cyanobacteria (Benzerara et al., 2022). Comparative genomics approaches such as genome‐wide association studies consist in sequencing the genomes of large numbers of microbial strains from the environment and comparing these genomes to identify genomic elements statistically associated with a given trait (Kobras et al., 2021). Here, the difficult relies in identifying a large enough number of bacterial strains displaying a similar mineral phenotype. Isolating microbes can be difficult and time‐consuming, and not all bacteria are amenable to culturing. But isolation is only the first step. Grouping isolates by mineral phenotype requires systematically testing their biomineralization capacity in the laboratory and then thoroughly characterizing the properties of their biomineral products. Unfortunately, biomineralization capability is not systematically assessed when a new microbial strain is described, and there is no doubt that many unsuspected biomineralizers are already sitting in strain collections. As an example of this, in 2012, the first cyanobacterium capable of intracellular carbonate biomineralization was described (Couradeau et al., 2012). Two years later, a microscopy re‐investigation of 68 cyanobacterial strains from culture collections revealed that seven of them could form intracellular carbonate biominerals (Benzerara et al., 2014). Thirteen additional strains were recently found by searching for genes associated with intracellular calcification in publicly available genomes (Benzerara et al., 2022). So, why did not we know about the biomineralization capabilities of these previously isolated strains? Biominerals may not always be obvious during observations of microbial cells under an optical microscope, especially when they are small, nanoparticulate inclusions. Fixation and dehydration techniques employed for the preparation of samples for electron microscopy may also result in the dissolution or alteration of the biominerals (e.g. Li et al., 2016).

Systematically screening newly isolated strains using mineral characterization methods that require no or minimal sample preparation steps such as Raman (e.g. Nims et al., 2019) or Fourier‐Transform Infrared Spectroscopy (e.g. Mehta et al., 2022) may in the future greatly increase the rate at which new biomineralizing strains are identified. For now, it is impossible to determine how many biomineralizing bacteria remain to be discovered. As of today, we are actually still discovering entirely new types of microbial biomineralization processes. The first bacterium capable of controlled intracellular biomineralization of silica was, for instance, only discovered last year (Li et al., 2022) (previously, biosilicification was thought to be uniquely present in Eukaryotes). The development of culture‐independent approaches will also certainly accelerate this discovery process. Here again, researchers studying magnetotactic bacteria are a few years ahead, due to the relative ease with which these bacteria can be recovered from the environment, using an applied magnetic field (Busigny et al., 2022; McCausland & Komeili, 2020). Single‐cell sequencing can now be performed on biomineralizing cells identified in environmental samples (e.g. Kolinko et al., 2016; Mansor et al., 2015), and newly developed methodological approaches are being developed for single‐cell genomics of environmental bacteria identified based on phenotypic characteristics using Raman‐assisted cell sorting (e.g. Song et al., 2017). The future application of such methods for culture‐independent studies of biomineralization systems should greatly improve our understanding of the genetic controls of microbial mineral phenotypes.

## CAN NEW MINERAL MATERIALS BE EVOLVED?

As we have seen, the development of biomineral screening approaches will in the future help us identify genes associated with certain mineral phenotypes in bacteria. Through rational design, this newly gained knowledge will help us engineer strains with improved biomineral properties for specific industrial applications. But what if we could entirely bypass these laborious genetic investigations, using directed evolution?

Evolutionary engineering has proven a powerful method to obtain microbial strains with desirable functional traits for various industrial applications, even in the case of microbes unamenable to genetic manipulation and/or for which there was no prior knowledge of the genotype–phenotype relationship for the trait of interest (Fernández‐Cabezón et al., 2019). Mimicking life's own mechanism for innovation and optimization through mutations and natural selection, this method consists in generating genetic diversity in a microbial strain, then applying a screen to select mutants displaying an improved phenotype. Iterations of the mutation‐selection cycle are repeated until the desired phenotypic outcome is obtained.

Modern directed evolution protocols now focus on the laboratory evolution of single genes rather than of whole microbial cultures (Arnold, 2018; Packer & Liu, 2015). Similarly, protocols for the bioengineering of minerals through molecular diversification and screening have been implemented (Bawazer, 2013; Rouge et al., 2011). These studies are based on synthetic biology systems in which only one gene of interest (e.g. coding for silicateins or ferritins; Bawazer et al., 2012; Liu et al., 2016) or one specific type of biomolecule (e.g. polypeptides; Lenders et al., 2017) are evolved. However, biomineral properties are likely to be under the simultaneous influence of many different genes, so that desirable mineral phenotypes may only emerge from non‐linear combinations of several mutations. For this reason, whole‐cell directed evolution is likely to yield the best results for the engineering of advanced mineral materials.

Screening is a keystone of directed evolution approaches, so it is not surprising that biomineral whole‐cell evolutionary engineering has to date only be attempted with magnetotactic bacteria (Tay et al., 2019). Hopefully, the development of biomineral screening tools such as those discussed previously in this article may accelerate the deployment of this approach for the directed evolution of other biomineral types.

## MINERAL MATERIAL BIOMANUFACTURING: A LOOK INTO THE FUTURE?

Imagine a factory. It does not emit any fume or smoke, and it is strikingly clean and silent. Along its walls are lined up massive bioreactors. The microbes they contain are continuously fed with seawater or local wastewater, supplemented with recycled metals and nutrients. They are growing advanced, engineered minerals, which will later be harvested and turned into batteries composites, metallic catalysts, or integrated into sophisticated biomedical devices. Some of the bioreactors have glass walls. Inside, bacteria are using sunlight to convert CO_2_ captured from the atmosphere into biomineralizing biomass. In this future, humans have successfully transitioned from an economical model based on fossil energies and petrochemical industries to a circular bioeconomy in which microbes are helping us meet our material needs without compromising Earth's resources. Of course, we are still a long way from materializing this vision, but these are exciting times for environmental microbiologists and geomicrobiologists, who have historically been leading research on microbes' complex interactions with their mineral environment. By working hand‐in‐hand with bioengineers and material scientists, they will develop more effective biomineral screening methods, improve our molecular‐level understanding of biomineralization mechanisms and develop scalable processes for mineral bioproduction. With these interdisciplinary efforts will emerge a new field of science and technology, which still remains to be named (‘biomaterials’ and ‘geobiotechnology’ being already taken, and designating very different concepts). Its mantra will be ‘if it can't be made (sustainably), it must be grown’.

## AUTHOR CONTRIBUTIONS

**Julie Cosmidis:** Conceptualization (lead); funding acquisition (lead); writing – original draft (lead); writing – review and editing (lead).

## CONFLICT OF INTEREST STATEMENT

The author has no conflict of interest.
